# CD8^+^ T cells are necessary for improved sepsis survival induced by CD28 agonism in immunologically experienced mice

**DOI:** 10.3389/fimmu.2024.1346097

**Published:** 2024-04-03

**Authors:** Jerome C. Anyalebechi, Yini Sun, Carolyn Davis, Maylene E. Wagener, Zhe Liang, Eileen M. Burd, Craig M. Coopersmith, Mandy L. Ford

**Affiliations:** ^1^ Department of Surgery, Emory University School of Medicine, Atlanta, GA, United States; ^2^ Emory Critical Care Center, Emory University School of Medicine, Atlanta, GA, United States; ^3^ Department of Critical Care Medicine, The First Affiliated Hospital of China Medical University, China Medical University, Shenyang, China; ^4^ Department of Pathology and Laboratory Medicine, Emory University, Atlanta, GA, United States; ^5^ Emory Transplant Center, Emory University School of Medicine, Atlanta, GA, United States

**Keywords:** T lymhpocyte, sepsis, regulatory T Cell, costimulation, memory T cell

## Abstract

**Introduction:**

A hallmark of T cell dysregulation during sepsis is the downregulation of costimulatory molecules. CD28 is one of T cell costimulatory molecules significantly altered on memory T cells during sepsis. We recently showed that treatment with a αCD28 agonist in septic immunologically experienced mice led to improved survival. Therefore, here we aimed to identify the cell subset(s) necessary for the survival benefit observed in the context of CD28 agonism, and to further investigate the mechanism by which CD28 agonism improves sepsis survival in immunologically experienced mice. Methods: Mice received specific pathogen inoculation to generate memory T cell populations similar in frequency to that of adult humans. Once these infections were cleared and the T cell response had transitioned to the memory phase, animals were rendered septic via cecal ligation and puncture in the presence or absence of an agonistic anti-CD28 mAb.

**Results:**

Results demonstrated that CD8^+^ T cells, and not bulk CD4^+^ T cells or CD25^+^ regulatory T cells, were necessary for the survival benefit observed in CD28 agonist-treated septic immunologically experienced mice. Upon examination of these CD8^+^ T cells, we found that CD28 agonism in septic immunologically experienced mice was associated with an increase in Foxp3^+^ CD8^+^ T cells as compared to vehicle-treated controls. When CD8^+^ T cells were depleted in septic immunologically experienced mice in the setting of CD28 agonism, a significant increase in levels of inflammatory cytokines in the blood was observed.

**Discussion:**

Taken together, these results indicate that CD28 agonism in immunologically experienced mice effectively suppresses inflammation via a CD8^+^-dependent mechanism to decrease mortality during sepsis.

## Introduction

Sepsis is defined as life-threatening organ dysfunction due to a dysregulated host response to infection (1). Immunosuppression in part through T cell dysregulation is a critical component of continued pathology during sepsis and eventual mortality (2). Mechanisms by which patients become immunosuppressed during sepsis include upregulation of inhibitory molecules, expansion of the regulatory T cell population, and downregulation of CD28 and HLA-DR-mediated activation pathways (2, 3). CD28 is a T cell costimulatory receptor which, upon ligation with CD80/CD86, transmits a costimulatory signal important for T cell activation, proliferation, and pro-inflammatory responses (4), making it a potential target for sepsis immunomodulatory therapy. Furthermore, there is evidence of significantly decreased expression of CD28 and its stimulatory ligands, CD80/CD86, in human septic patients (2).

Given this possible role of CD28 in sepsis immunosuppression, multiple studies have found that modulation of CD28 and its ligands impact the emergence of immune dysregulation during sepsis, and thus impact mortality. One study found that deletion of CD80 or CD86 decreased inflammatory cytokine production during murine cecal ligation and puncture (CLP) and improved mortality (5). Similar findings were shown with combined blockade or deletion of CD80/86 in the same murine model of sepsis (6). Further evidence of interruption of the CD28/B7 complex through use of a B7-2 and CD28 dimer interface mimetic peptides prevented superantigen binding to the CD28/B7 complex and attenuated inflammatory cytokines (7). A peptide antagonist directed just to CD28 showed similar results and improved survival in a murine model of necrotizing soft-tissue infection (8). In contrast, another study showed antibody-mediated CD28 stimulation could be protective for intestinal immune responses during sepsis due to its role in T cell transendothelial migration (9).

Given these somewhat conflicting results, our group recently investigated the role of CD28 costimulatory signaling during sepsis through the use of an agonistic αCD28 Ab (10). Additionally, given that CD28 expression is altered on memory T cells vs. naïve T cells in both mice and humans, and given that laboratory mice possess significantly fewer memory T cells than adult humans (11), we utilized our novel “memory mouse” model in which mice receive specific pathogen inoculation to generate memory T cell populations similar in frequency to that of adult humans (12). This allowed us to also investigate the role of CD28 in a more physiologically relevant murine sepsis model. We found that in naïve septic mice, αCD28 Ab treatment resulted in worsened mortality, but in the immunologically experienced septic mice, a significant survival advantage was observed. This was associated with reduced T cell apoptosis in septic immunologically experienced mice but not in the naïve septic mice (10).

Here, we investigated the mechanisms underlying the survival advantage observed in immunologically experienced mice treated with agonistic anti-CD28. We found that CD8^+^ T cells, and not regulatory T cells or CD4^+^ conventional T cells (Tconv), are necessary for the effect. Depletion of CD8^+^ T cells increased both systemic inflammation and mortality during sepsis in the immunologically experienced host.

## Materials and methods

### Generation of “immunologically experienced mice”

Six-week-old male and female C57BL/6J mice were obtained from Jackson Laboratories (Bar Harbor, ME) for use in all experiments. 6-8 wk old mice were first infected via intraperitoneal injection with 1x10^4^ CFU of *Listeria monocytogenes* diluted in 0.5 mL of PBS. This was followed by infection with 2x10^5^ PFU of lymphocytic choriomeningitis virus (LCMV, Armstrong strain) diluted in 0.5 mL of R10 media, delivered via intraperitoneal injection 30 days later. All mice then underwent CLP 60 days after the initial infection.

### Cecal ligation and puncture (CLP)

While mice were under monitored anesthesia, a midline incision was made. The cecum was identified, ligated with silk suture, and a 25-gauge needle was used to puncture the cecum twice. Stool was released from the punctured cecum into the abdomen. All abdominal contents were returned to the abdomen prior to closing the incision with vicryl suture. For pain control, all mice received buprenorphine (0.1 mg/kg) and bupivacaine (0.25 mg/kg) via subcutaneous injection prior to CLP. Additionally, immediately following CLP, all mice received 1 mL of normal saline and antibiotics (50 mg/kg of ceftriaxone and 35 mg/kg of metronidazole) delivered via subcutaneous injection. Three further doses of antibiotics were administered at 12, 24, and 36 hours after CLP for a total of 4 doses. Mice were either monitored for 7-day survival or euthanized at 24 hours after CLP.

### Antibody administration

αCD4 antibody (BioXCell, GK1.5) was used for CD4 T cell depletion. αCD8 antibody (BioXCell, clone YTS 196.4) was used for CD8 T cell depletion. αCD25 antibody (BioXCell, clone PC-61.5.3) was used for regulatory T cell depletion. All depletion antibodies were administered 24 hours prior to CLP and again 24 hours after CLP. Depletion was verified in the spleen at 24h (**Supplementary Figure 1**). The agonistic αCD28 antibody (BioXCell, clone 37.51, Syrian hamster IgG) was given immediately following CLP and again on post-operative days 2, 4, and 6.

### Flow cytometry

24hrs after CLP, mice were euthanized, and their spleens were harvested for processing into single-cell suspensions using a 70 μm filter. Splenocytes were rinsed and resuspended in 10 mL of PBS with 200 μL of each sample placed into a 96-well plate for staining. Anti-CD3 (BioLegend, clone 17A2), anti-CD8 (BD, clone 53-6.7), anti-CD4 (Invitrogen, clone RM4-5), anti-CD44 (BioLegend, clone IM7), anti-TIGIT (BD, clone 1G9), anti-PD-1 (BioLegend, clone 29F.1A12), anti-CD25 (BioLegend, clone PC61), anti-CD69 (BioLegend, clone H1.2F3), and anti-LAP (Thermo Fisher Scientific, clone TW7-16B4) were used for cell surface immunophenotyping. Anti-Foxp3 (Invitrogen, clone FJK-16s) was used for identification of regulatory T cells after splenocytes were fixed and permeabilized (Foxp3 kit, BD Biosciences). CountBright™ Absolute Counting Beads (Thermo Fisher Scientific) were added to samples after staining to calculate the absolute numbers of cells per spleen. Samples were run on a Fortessa flow cytometer (Becton Dickinson) and data was analyzed using FlowJo software (ver. 10.8.1).

### Intracellular cytokine staining

20 μg/mL PMA and 1 μg/mL of ionomycin were used to stimulate splenocytes in the presence of GolgiStop (BD Biosciences). The cells were then fixed and permeabilized using a Foxp3 kit (BD Biosciences). Intracellular cytokine staining was then performed using anti- IL-2 (BioLegend, clone JES6-5H4), anti-IL-10 (BioLegend, clone JES5-16E3), anti-IFNγ (BD, clone XMG1.2), anti-TNF (BioLegend, clone MP6-XT22), anti-IL-6 (Invitrogen, clone MP5-20F3), and anti-IL-1β (Invitrogen, clone NJTEN3).

### Serum cytokines

24 hours after CLP, mice were euthanized, and whole blood was obtained via cardiac puncture. The samples were then centrifuged at 10,000 rpm at 4°C and serum was obtained. Cytokine concentrations of IL-1β, IL-6, MCP-1, IFN-γ, IL-2, and IL-10 were measured on 50 μLof serum diluted 1:4 in PBS using the Bio-Plex 200 System with all samples run in duplicate. Data were analyzed using the Bio-Plex Manager 3.0 software.

### Blood and peritoneal fluid bacterial cultures

Whole blood was obtained via cardiac puncture 24 hours after CLP. Simultaneously, 3mL of sterile saline was used as a peritoneal lavage to obtain peritoneal fluid (PF) samples. 100 μL of whole blood and 100 μL of PF were then taken to detect bacterial load. The samples were serially diluted in sterile saline and cultured on sheep’s blood agar plates (Remel) overnight at 37°C in 5% CO_2_. Colony counts were then determined on plates which had received 1:10^4^ diluted inoculum.

### Statistics

The log-rank test was used for statistical analysis of survival curves. Survival experiments are pooled from 3 independent experiments with a total of 10-20 mice/group as indicated. Analyses of cellular compartments is pooled from 2 independent experiments with a total of 9-10 mice/group as indicated. Analyses of blood and peritoneal fluid cultures included data pooled from 2 independent experiments with a total of 10 mice/group. The Mann-Whitney U test was used for all other analyses. A *p* value of less than or equal to 0.05 was considered significant. All analyses were performed using GraphPad Prism 9.

### Study approval

This study was conducted under approval from the Emory University Institutional Animal Care and Use Committee (protocol number 201700378).

## Results

### CD8^+^ T cells are necessary for the survival benefit observed in septic CD28 agonist-treated immunologically experienced mice

**Figure 1:**
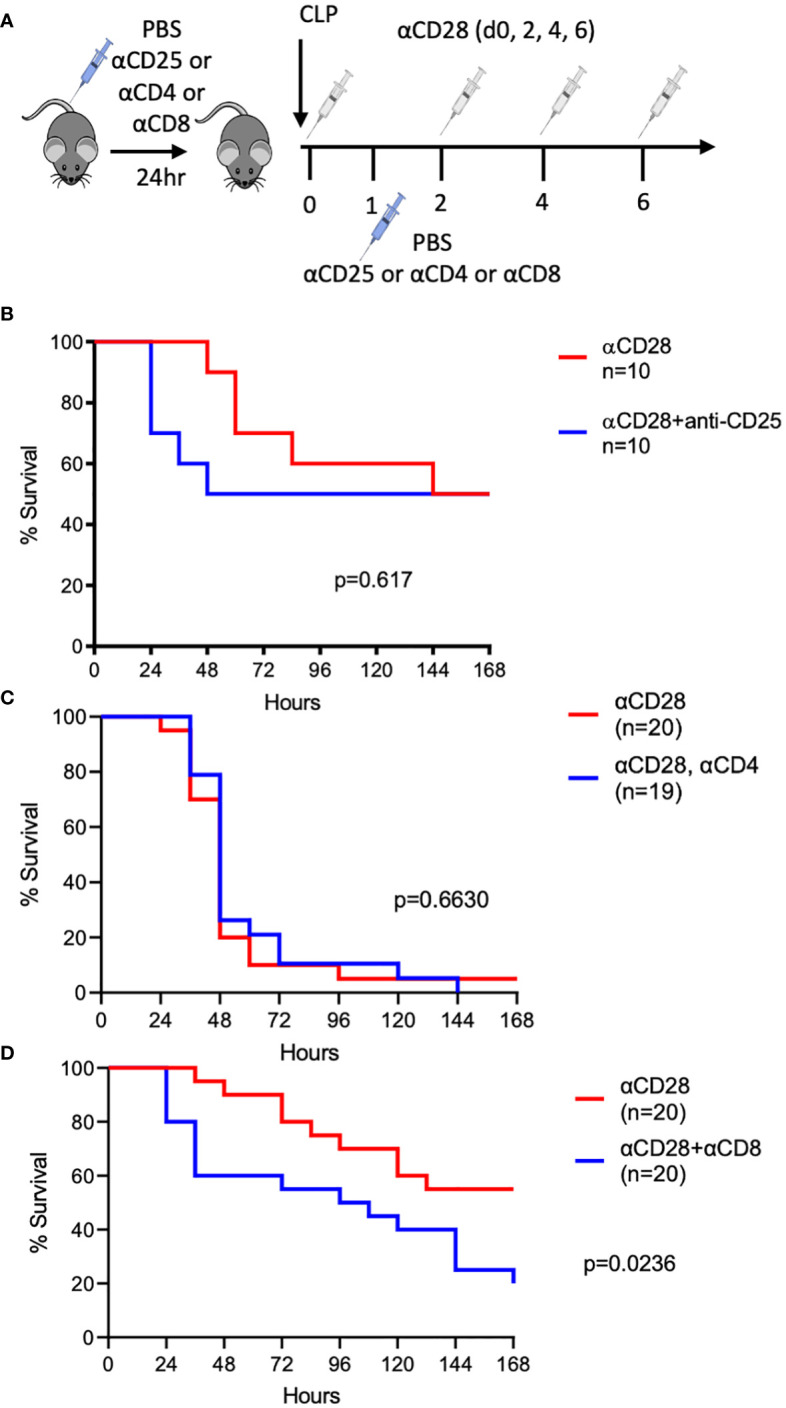
Depletion of CD8^+^, but not CD4^+^ or CD25^+^, T cells worsens the survival of CD28-agonist treated immunologically-experienced septic mice. **(A)** Experimental design. Immunologically experienced mice underwent CLP and received anti-CD28 Ab on days 0, 2, 4, and 6 (white syringes) and either anti-CD25, andti-CD4, or anti-CD8 on days -1 and 1 (blue syringes). **(B)** Immunologically experienced mice underwent CLP and received anti-CD28 Ab and either anti-CD25 (n=10) or PBS (n=10). **(C)** Immunologically experienced mice underwent CLP and received anti-CD28 Ab and either anti-CD4 (n=19) or PBS (n=20). **(D)** Immunologically experienced mice underwent CLP and received anti-CD28 Ab and either anti-CD8 (n=20) or PBS (n= 20). Mice were monitored for 7-day survival. Data are pooled from 3 independent experiments with a total of 10-20 mice/group as indicated. Mantel-Cox log-rank test was used to determine significance. A *p* value of less than or equal to 0.05 was considered significant.

We previously demonstrated that CD28 agonism results in improved sepsis survival in immunologically experienced, but not naïve, hosts (10). To determine the immune cell subset(s) that impact sepsis survival in the context of CD28-agonist treatment in immunologically-experienced septic mice, depleting antibodies against CD25, CD4, or CD8 (or control PBS) were administered before and after CLP in mice receiving anti-CD28 injections (**Figure 1A**). We previously reported that an isotype control for the CD28 agonist had no effect on sepsis survival (10). Despite the fact that we previously reported an increase in IL-10 secretion from CD4^+^ Foxp3^+^ Treg in septic anti-CD28-treated immunologically experienced mice (10), no difference in 7-day survival was observed when Tregs were depleted in the setting of CD28 agonism during sepsis (50% vs. 50%, *p=0.617*, **Figure 1B**), indicating that Tregs did not modify sepsis survival under these conditions. Similarly, CD4-depleted animals exhibited no difference in sepsis survival in the context of CD28 agonism (5% vs. 0%, *p=0.663*, **Figure 1C**). However, significantly worsened survival was observed when animals were depleted of CD8^+^ T cells in the context of CD28 agonism (55% vs. 20%, *p=0.024*, **Figure 1D**). These data demonstrate that CD8^+^ T cells critically impacted sepsis survival following treatment with a CD28 agonist in septic immunologically experienced mice.

### CD28 agonism in septic immunologically experienced mice results in increased CD44^hi^ CD8^+^ T cells

**Figure 2:**
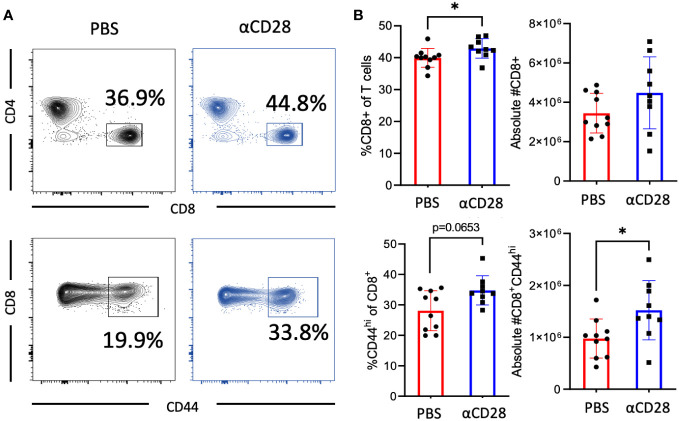
αCD28-agonist treatment accrues CD44^hi^ memory T cells following CLP in immunologically-experienced mice. B6 Immunologically experienced mice were rendered septic by CLP. αCD28 (n=9) or PBS (n=10) was administered immediately following surgery. Mice were sacrificed 24hr after surgery, spleens were harvested, and CD8 and CD44 expression were determined by flow cytometry. **(A)** Representative flow plots and **(B)** summary data depicting frequencies and absolute numbers of Increased expression of bulk and CD44^hi^ CD4^+^ and CD8^+^ T cells in vehicle vs αCD28-treated animals. Data are pooled from 2 independent experiments with a total of 9-10 mice/group as indicated. Significance was determined by Mann Whitney U test. A *p* value of less than or equal to 0.05 was considered significant. *p<0.05.

Given the identification of CD8^+^ T cells as a critical cell subset underlying the ability of the CD28 agonist to improve sepsis survival, we next queried the effect of CD28 agonism on the CD8^+^ T cell compartment in memory septic mice. Immunologically experienced mice which had received the agonistic CD28 antibody, as well as mice which had received the vehicle control, underwent CLP. Animals were sacrificed 24hrs later and spleens were harvested for assessment of the impact of CD28 agonism during sepsis on the T cell compartment (gating strategy shown in **Supplementary Figure 2A**). Results indicated a significant increase in the frequency of bulk CD8^+^ T cells in mice treated with the CD28 agonist as compared to vehicle-treated controls (42.6% vs. 40.2%, *p=0.017*, **Figures 2A, B**). Furthermore, results also showed an increase in the number (and a trend toward an increase in the frequency) of CD44^hi^ CD8^+^ T cells in animals treated with CD28 agonism vs. vehicle controls (1.4x10^6^ vs. 1.0x10^6^ cells/spleen, *p=0.017*, **Figures 2A, B**). Given these results, it is interesting to note that CD44^hi^ CD8^+^ T cells exhibit a significant increase in CD28 expression level as compared to CD44^lo^ CD8^+^ T cells in memory septic mice (*p<0.0001*, **Supplementary Figure 2B**).

### CD28 agonism did not affect markers of activation, proliferation, or exhaustion on CD8^+^ T cells

**Figure 3:**
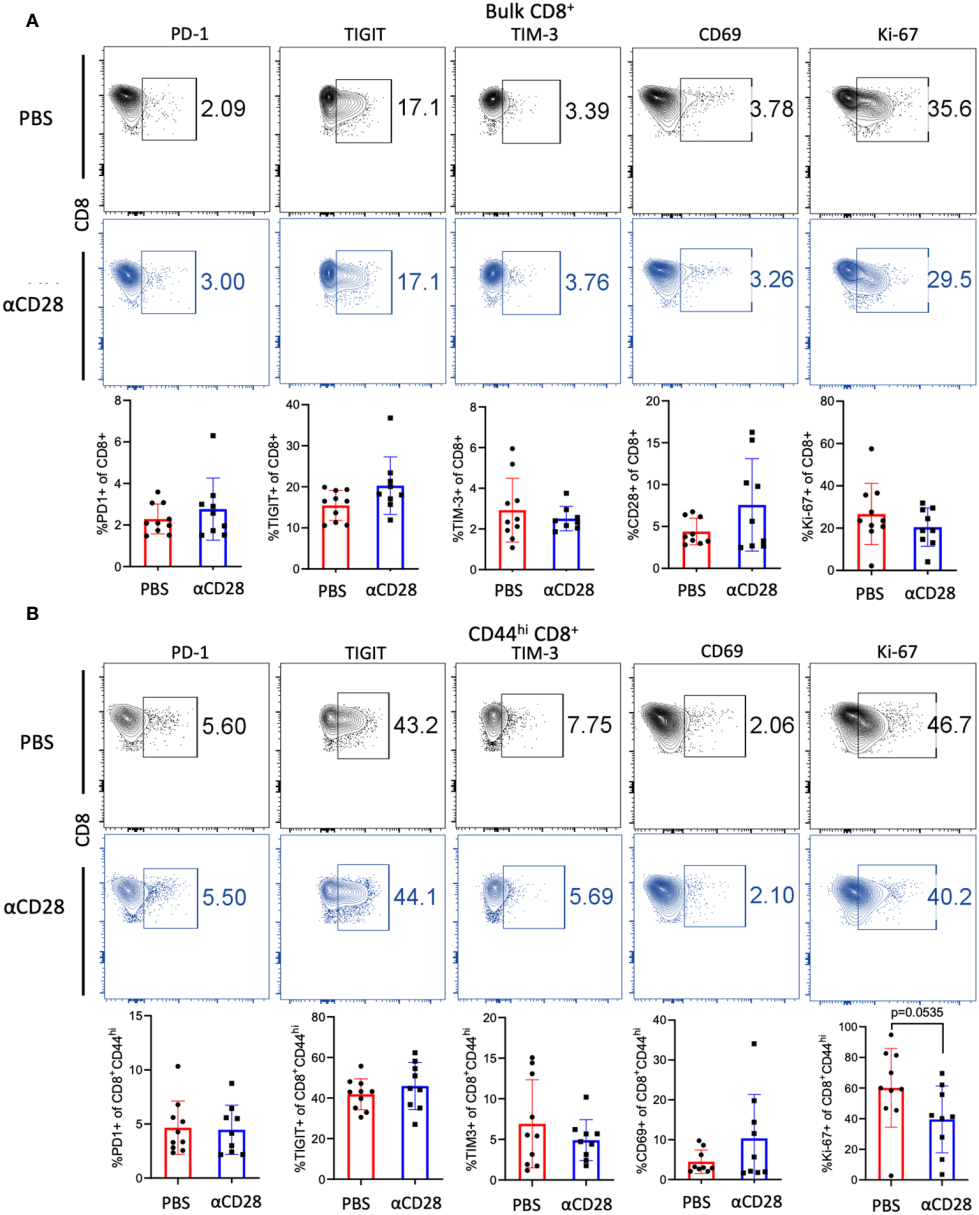
αCD28-agonist treatment does not affect markers of exhaustion, activation, or proliferation of CD8^+^ T cells, in immunologically-experienced septic mice. B6 Immunologically experienced mice were rendered septic by CLP. αCD28 (n=9) or PBS (n=10) was administered immediately following surgery. Mice were sacrificed 24hr after surgery, spleens were harvested, and expression of activation, exhaustion, and proliferation markers were determined by flow cytometry. **(A)** CD28 agonism during CLP did not change the expression of the indicated markers on bulk CD8^+^ T cells **(B)** CD28 agonism during CLP did not change the expression of the indicated markers on CD44^hi^ CD8^+^ T cells. Data are pooled from 3 independent experiments with a total of 9-10 mice/group as indicated. Significance was determined by Mann Whitney U test. A *p* value of less than or equal to 0.05 was considered significant.

To determine if the observed increase in the CD44^hi^ CD8^+^ T cell compartment was the result of increased proliferation and/or reduced exhaustion, the level of expression of activation markers, coinhibitory/exhaustion markers, and a marker of proliferation, Ki-67, in immunologically experienced mice which had received αCD28 vs. vehicle control during CLP was assessed. No difference in expression of exhaustion or early activation markers in either bulk or memory CD8^+^ T cell compartments were observed (**Figures 3A, B**). Bulk CD8^+^ T cells isolated from PBS vs. αCD28-treated septic immunologically experienced mice exhibited no difference in the frequency of Ki-67^+^ cells, however, CD44^hi^ CD8^+^ T cells isolated from anti-CD28 treated-mice exhibited a trend towards decreased Ki-67 expression relative to cells isolated from-PBS-treated mice (41.2% vs. 59.7*%, p=0.0535*, **Figure 3B**).

### CD8^+^ T cells producing inflammatory cytokines were not increased following CD28agonism

**Figure 4:**
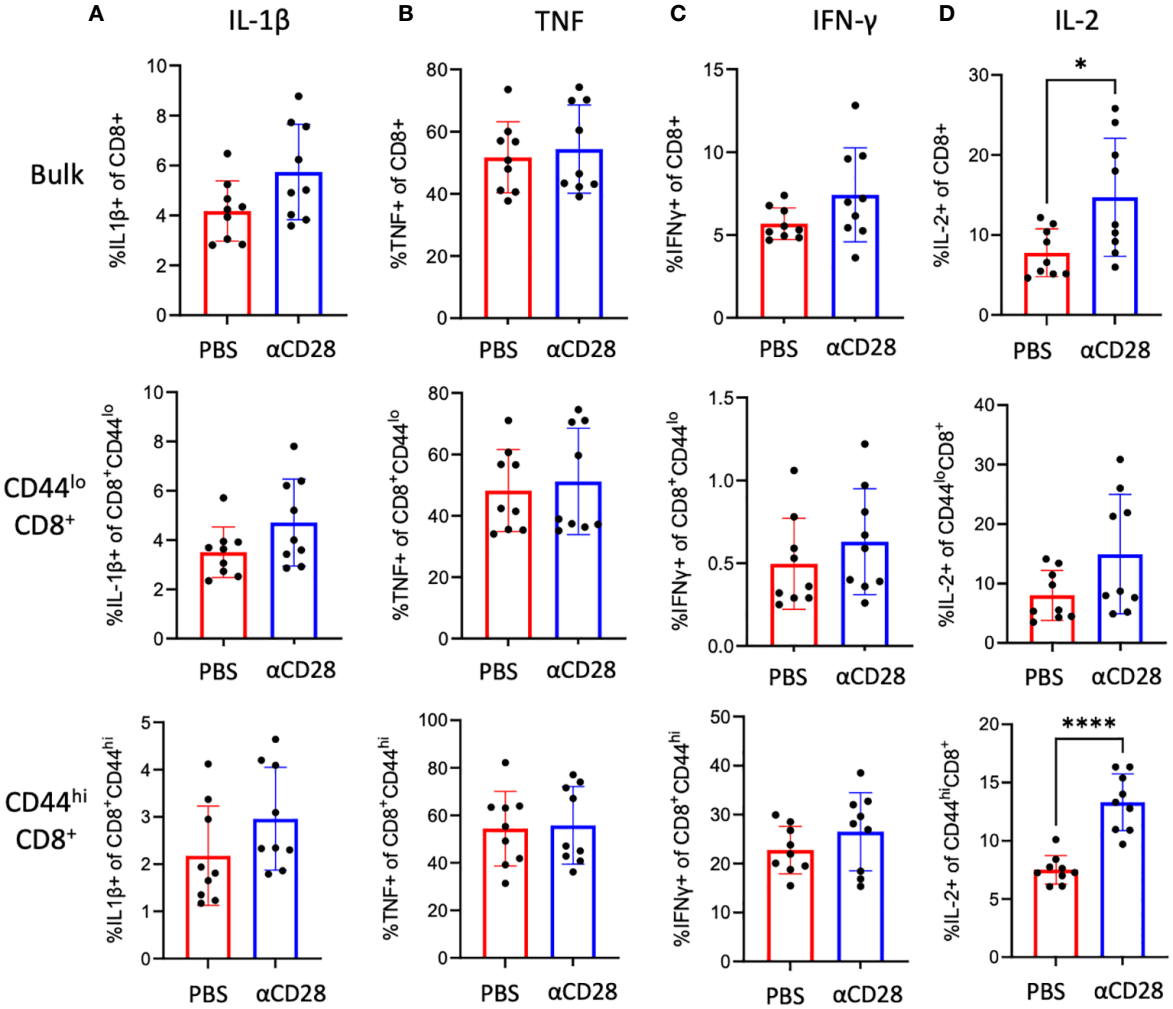
αCD28-agonist treatment significantly enhances IL-2 responses in CD8^+^ T cells, in immunologically-experienced septic mice. B6 Immunologically experienced mice were rendered septic by CLP. αCD28 (n=9) or PBS (n=10) was administered immediately following surgery. Mice were sacrificed 24hr after surgery and spleens were harvested. Intracellular IL-1β **(A)**, TNF **(B)**, IFN-γ **(C)**, and IL-2 **(D)** expression was determined on bulk CD8^+^, CD44^lo^ CD8^+^, and CD44^hi^ CD8^+^ by flow cytometry following ex vivo restimulation with PMA/ionomycin. Data are pooled from 3 independent experiments with a total of 9 mice/group as indicated. Significance was determined by Mann Whitney U test. A *p* value of less than or equal to 0.05 was considered significant. *p<0.05. ****p<0.0001.

Although there were no differences in activation or exhaustion immunophenotype of CD8^+^ T cells isolated from anti-CD28 treated septic immunologically experienced mice, given the fact that CD28 costimulation can impact T cell effector function and cytokine production (13), we questioned whether administration of the αCD28 Ab during sepsis led to changes in cellular function as evidenced by intracellular cytokine production following *ex vivo* stimulation with PMA/ionomycin. Unstimulated samples were used as negative controls. No differences were observed in the frequencies of IL-1β- (**Figure 4A**), TNF (**Figure 4B**) or IFN-γ-producing (**Figure 4C**) bulk, CD44^lo^, or CD44^hi^ CD8^+^ T cells between animals that received αCD28 Ab vs. vehicle control. We observed a significant increase in IL-2 production in bulk CD8^+^ T cells in animals that received αCD28 Ab vs vehicle control (6.6% vs. 11.3%, *p=0.040*, **Figure 4D**). This was driven primarily by memory CD44^hi^ CD8^+^ T cells (7.3% vs. 13.3%, *p<0.0001*, **Figure 4D**).

### CD28 agonism resulted in an increase in CD8^+^ Foxp3^+^ T cells

**Figure 5:**
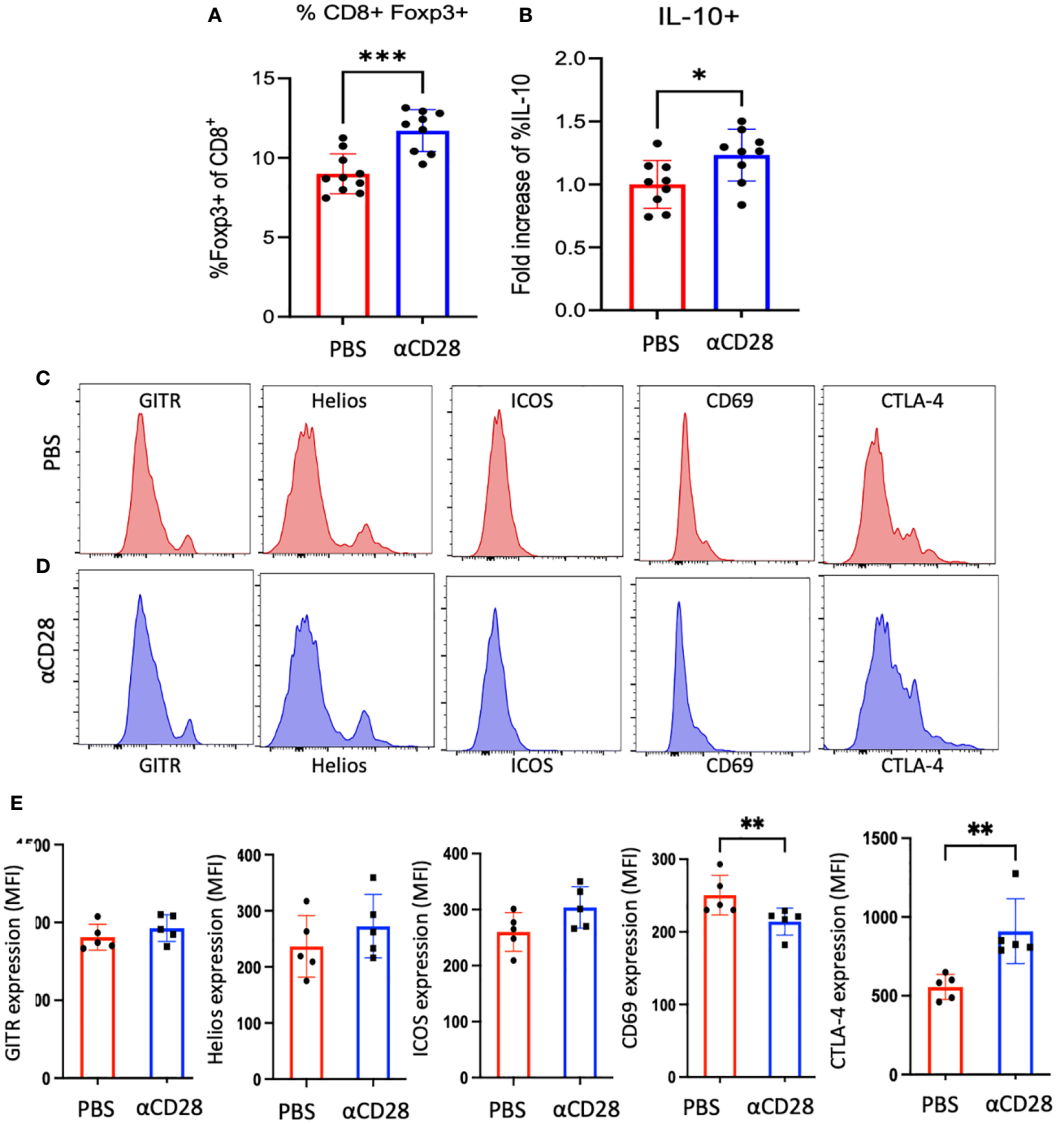
αCD28-administration promotes Foxp3^+^ CD8^+^ T cell accumulation in immunologically-experienced septic mice. B6 Immunologically experienced mice were rendered septic by CLP. αCD28 (n=9) or PBS (n=10) was administered immediately following surgery. Mice were sacrificed 24hr after surgery and spleens were harvested and stained for Foxp3. Intracellular cytokine expression was determined by flow cytometry following ex vivo restimulation with PMA/iono. **(A, B)** Summary data showing that CD28 agonism during sepsis significantly increased the frequency of Foxp3^+^ CD8^+^ T cells, and Foxp3^+^ CD8^+^ exhibited increased IL-10 expression with CD28 agonism. Data are pooled from 3 independent experiments with a total of 9-10 mice/group as indicated. **(C-E)** Phenotypic analysis of Foxp3^+^ CD8^+^ T cells isolated from vehicle vs CD28 agonist-treated mice. Data are pooled from 2 independent experiments with a total of 5 mice/group. Significance was determined by Mann Whitney U test. A *p* value of less than or equal to 0.05 was considered significant. *p<0.05, **p<0.01, ***p<0.001.

Previous studies have identified Foxp3-expressing CD8^+^ T cells as indicative of a distinct suppressor CD8^+^ T cell subset (14–17). Upon interrogation of the presence of Foxp3^+^ CD8^+^ T cells immunologically experienced septic animals, we observed a significant increase in the proportion of CD8^+^ Foxp3^+^ T cells in septic immunologically experienced mice treated with CD28 agonist as compared to animals treated with vehicle alone (12.1% vs. 8.7% respectively, *p=0.001*, **Figure 5A**). Gating strategy is shown in **Supplementary Figure 3A**. In vehicle control-treated mice, ~18% of Foxp3^+^ CD8^+^ T cells exhibited expression of CD28 (**Supplementary Figure 3B**). Anti-CD28-treated animals in which CD28 staining is blocked are shown as a negative control. Furthermore, in septic immunologically experienced mice treated with CD28 agonist, Foxp3^+^ CD8^+^ T cells exhibited a significant increase in IL-10 production (1.26-fold increase, *p=0.0315*, **Figure 5B**). Analysis of the phenotypic characteristics of these cells revealed a significant decrease in the expression of activation marker CD69 on Foxp3^+^ CD8^+^ T cells in animals treated with CD28 agonist vs vehicle control, along with a significant increase in the expression of CTLA-4, which may be indicative of an increase in suppressor function in these cells. No change was observed in the expression of GITR, Helios, or ICOS (**Figures 5C–E**).

### Depletion of CD8^+^ T cells in the setting of CD28 agonism led to increased systemic inflammatory cytokines

**Figure 6:**
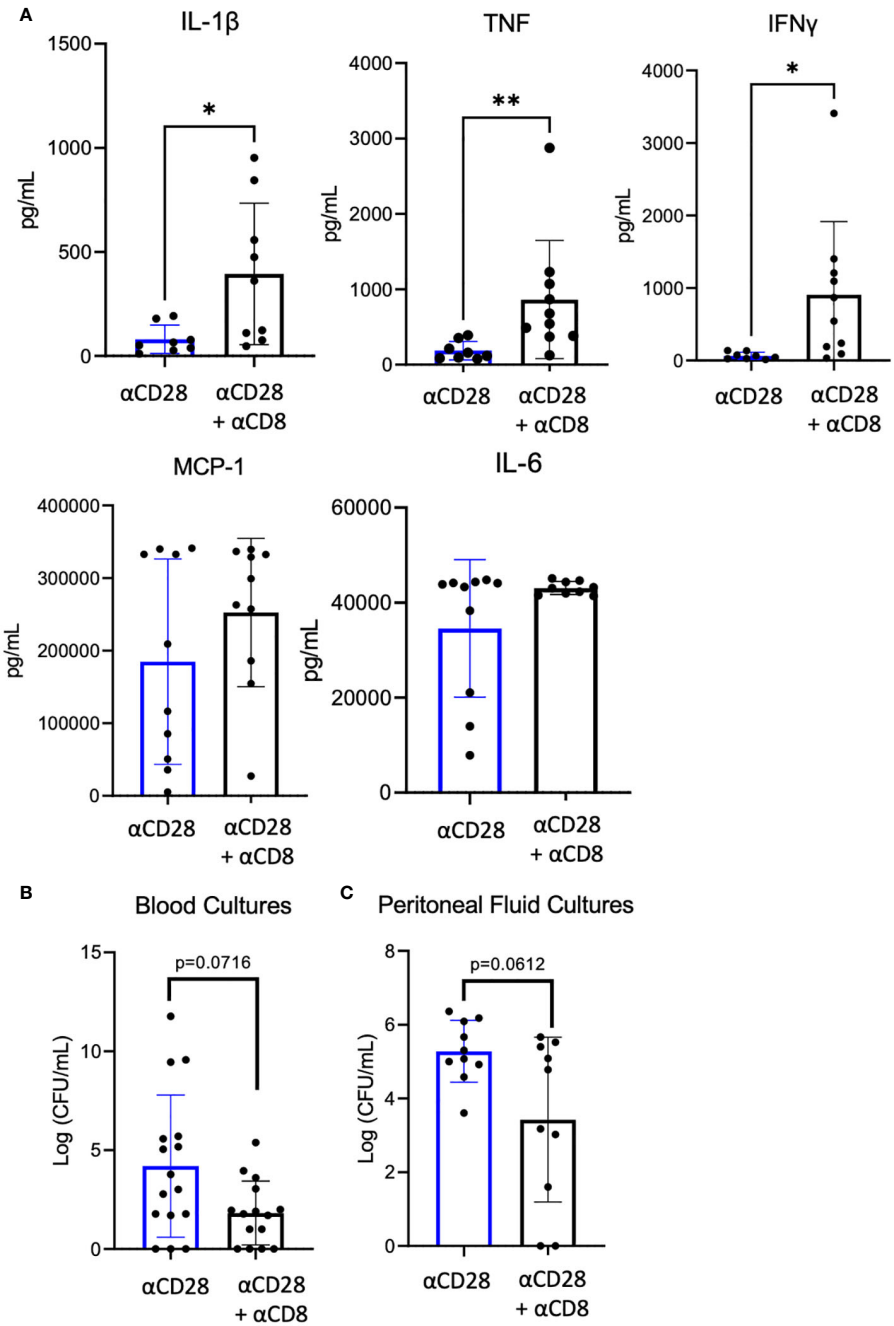
Significant increase of serum IL-1β, TNF, and IFN-γ after αCD28-agonist treatment in immunologically-experienced septic mice depleted of CD8^+^ T cells. αCD8 (n=10) or PBS (n=10) was administered to B6 Immunologically experienced mice. They all were then rendered septic by CLP and given αCD28 immediately following surgery. Mice were sacrificed 24hr after surgery and whole blood was collected. **(A)** Serum cytokine concentrations. **(B)** Blood and peritoneal fluid bacterial load. Data are pooled from 2 independent experiments with a total of 10 mice/group. Significance was determined by Mann Whitney U test. A *p* value of less than or equal to 0.05 was considered significant. *p<0.05, **p<0.01.

Given the observed increase in IL-10^+^ CD8^+^ Foxp3^+^ T cells in septic immunologically experienced mice treated with anti-CD28 vs vehicle control, we next determined the impact of CD8^+^ T cell depletion on systemic inflammation in the setting of CD28 agonism in immunologically experienced mice during sepsis. To test this, we performed CLP on two groups of mice where both groups were administered αCD28 Ab but only one group underwent depletion of CD8^+^ T cells. Blood obtained from mice 24 hours after CLP revealed increased concentrations of the proinflammatory cytokines IL-1β (59 pg/mL vs. 361 pg/mL, *p=0.0206*), TNF (139 pg/mL vs. 610 pg/mL, p=0.0014), and IFNγ (55 pg/mL vs. 706 pg/mL, *p=0.0021*) in the CD8^+^ depletion groups as compared to non-CD8^+^-depleted controls (**Figure 6A**). Furthermore, depletion of CD8^+^ T cells resulted in a trend toward decrease bacterial load in both the blood and peritoneal fluid (**Figures 6B, C**). These data show that the increase in inflammatory cytokines was not due to increased bacterial load. Instead, they show that depletion of CD8^+^ T cells in CD28-agonist treated immunologically experienced mice results in increased immune activation and inflammation, and suggest that the CD8^+^ T cells in CD28 agonist-treated septic animals were functioning in an immunosuppressive manner to limit inflammation.

## Discussion

This study addressed the role of CD28 signaling during sepsis-induced immune dysregulation in immunologically experienced hosts. Results demonstrate a functional role of CD28 agonism during sepsis in immunologically experienced mice, which are likely more physiologically similar to immunologically experienced adult humans, as compared to naïve laboratory mice housed under specific pathogen-free conditions. More specifically, data indicate that memory CD8^+^ T cells following CD28 agonism during sepsis may function in an immunosuppressive manner, insofar as depletion of these cells leads to increased systemic inflammatory cytokines and a trend toward lower bacterial counts in both the blood and peritoneal fluid. This conclusion is also consistent with the observed increase in Foxp3^+^ CD8^+^ T cells (putative suppressor cells) under these conditions. The observation of both *increased* immunity (as measured by increased inflammatory cytokine levels and a trend toward decreased bacterial load) and *increased* mortality leads us to speculate that following the deletion of CD8^+^ T cells in immunologically experienced septic mice treated with agonistic anti-CD28, the mice are dying from an overexuberant inflammatory response, and not immune incompetence. It is also in line with our previously published data showing that the beneficial effect of CD28 agonism on sepsis survival in immunologically experienced hosts is dependent on IL-10 (10). Jointly with our previous findings, this current study indicates that CD28 agonism in immunologically experienced mice promotes the generation of an immunosuppressive environment that quells systemic inflammation and is beneficial for survival during sepsis.

As discussed above, reports regarding the role of CD28 signaling in sepsis have been somewhat contradictory. However, the preponderance of studies analyzing the role of CD28 and/or its ligands CD80/86 in models in which the recipients used are immunologically naïve have demonstrated that deleting or antagonizing CD28 signaling results in improved sepsis mortality. Importantly, in keeping in line with these studies, we recently showed that CD28 agonism was moderately deleterious in naïve septic hosts (10). In contrast, CD28 agonism conferred a significant survival benefit when administered to immunologically experienced septic hosts. Of note, this increased survival was associated with increased IL-10 production from CD4^+^ Foxp3^+^ Treg, and systemic blockade of IL-10 abrogated the protective effect of the CD28 agonist in septic immunologically experienced mice (10). Thus, in the previously published study we surmised that CD4^+^ Foxp3^+^ IL-10-producing cells could be mechanistically responsible for the observed increase in sepsis survival in CD28 agonist-treated animals (10). However, here we show that depletion of either CD4^+^ T cells or CD25^+^ T cells, widely used as a strategy to deplete CD25^+^ CD4^+^ Foxp3^+^ Treg, did not reverse the survival benefit of CD28 agonist in immunologically experienced septic mice. Instead, we observed that depletion of CD8^+^ T cells removed the survival benefit conferred by the CD28 agonist in immunologically experienced septic mice. Owing to the observation that CD8^+^ T cells in CD28 agonist-treated immunologically experienced septic mice exhibited increased IL-10 secretion and our published finding that IL-10 neutralization erases the benefit of CD28 agonism in immunologically experienced septic mice (10), we conclude that the immunoregulatory effect of IL-10-producing CD8^+^ Foxp3^+^ T cells contributes to the salutary effect of CD28 agonism during sepsis in immunologically experienced hosts.

Immunosuppressive Foxp3^+^ CD8^+^ T cells have been described in other studies. Conditions that result in the generation of suppressive CD8^+^ T cells (*in vivo* or *in vitro*) include continuous antigen stimulation with staphylococcal enterotoxin B (15), administration of anti-CD3 mAb (18), stimulation using autologous LPS-activated dendritic cells (19), and priming naïve CD8^+^ T cells with CD40 ligand-activated plasmacytoid dendritic cells (20). Most relevant to our study, suppressive CD8^+^ T cells have also been shown to be induced through increasing CD28 costimulatory signaling in the presence of TGF-β (16). Because TGF-β is significantly upregulated during sepsis (21), our results are consistent with the hypothesis that CD28 agonism during sepsis directly increases a population of immunosuppressive CD8^+^ Foxp3^+^ T cells (15–17). This hypothesis of a direct effect of CD28 signaling is consistent with our data showing that a subset of CD8^+^ Foxp3^+^ Treg are CD28^+^ under these conditions. However, many previous studies report suppressive CD8^+^ T cells to be CD28 negative (22, 23). It is also possible that the precursor to Foxp3^+^ CD8^+^ T cells may begin as CD28^+^ cells which are modified by CD28 signaling, but subsequently downregulate CD28 and upregulate Foxp3. Anti-CD28 antibody could also be impacting CD8^+^ Foxp3^+^ T cells indirectly by freeing up more CD80/CD86 for CTLA-4 ligation and signaling. This hypothesis is supported by our findings of a significant increase in CTLA-4 expression on CD8^+^ Foxp3^+^ following administration of the anti-CD28 mAb in septic immunologically experienced mice. These possibilities warrant further investigation. Of note, CTLA-4 expression was not different on bulk CD4^+^ or CD8^+^ T cells, nor on CD44^hi^ CD8^+^ or CD44^lo^ CD8^+^ T cells when comparing cells isolated from vehicle control-treated and anti-CD28-treated septic immunologically experienced mice (data not shown).

CD28 agonism during sepsis also led to increased IL-2 production from bulk CD8^+^ T cells, driven mainly by CD44^hi^CD8^+^ T cells (**Figure 4**). IL-2 is essential for generation, survival, and functional activity of Foxp3^+^ regulatory T cells (24), further supportive evidence that CD28 agonism could promote an immunosuppressive environment. The significance of the observed increase in IL-10 secretion from Foxp3^+^ CD8^+^ T cells during sepsis is based on studies showing that IL-10 can be both beneficial (25–27) and detrimental (28, 29) to survival during sepsis. In the setting of CD28 agonism, we previously observed that neutralization of IL-10 significantly worsened mortality in septic immunologically experienced mice (10), evidence to support the beneficial effect of immunosuppression observed in immunologically experienced mice with CD28 agonism.

While here we show that CD28 agonism promoted IL-10 production in CD8^+^ Foxp3^+^ T cells, we have previously shown that CD28 agonism promoted the proliferation and IL-10 production of traditional CD4^+^ Foxp3^+^ T cells, and functionally increased their suppressive capacity, in the context of immunologically experienced but not naive recipients (10). The mechanisms underlying these related findings are not clear, but could be related to differential effects of CD28 agonism on the recently-described memory Treg subset (30). Following exposure to antigen in the periphery, thymus-derived Treg become activated, proliferate and differentiate into more potent suppressors (30). Following the resolution of inflammation, a subset of these activated Treg can persist and function to suppress subsequent immune responses in a more accelerated, recall fashion when antigen is re-encountered. Extensive phenotypic and functional analysis of memory Treg has been hampered by a lack of specific phenotypic markers to define these cells in both human and mouse. Thus, further investigation is needed to dissect the role of CD28 signaling on memory Treg cells during sepsis. However, based on the results presented here as well as our previously published work, it is tempting to speculate that CD28 signaling may differentially impact naive and memory Treg cells, in a manner analogous to the differential requirements for CD28 costimulation on naive vs. memory conventional T cells (40, 41). One limitation of our study is that differences in the expression and regulation of CD28 on distinct T cell subsets in mice vs. humans may exist. Indeed, the results of the TGN141 study (31), in which agonistic anti-CD28 was administered to five healthy human volunteers who experienced catastrophic cytokine storm and which was not predicted from studies in animal (canine) models (32), may highlight differences in CD28 regulation or signaling under these conditions. Additional studies are therefore required to understand the role of CD28 on memory and regulatory T cell subsets in humans, both in health and in the context of immune dysregulation.

## Data availability statement

The raw data supporting the conclusions of this article will be made available by the authors, without undue reservation.

## Ethics statement

The animal study was approved by Emory University Institutional Animal Care and Use Committee. The study was conducted in accordance with the local legislation and institutional requirements.

## Author contributions

MF: Writing – review & editing, Writing – original draft, Visualization, Supervision, Project administration, Funding acquisition, Formal analysis, Data curation, Conceptualization. JA: Writing – review & editing, Writing – original draft, Visualization, Investigation, Formal analysis, Data curation. YS: Writing – review & editing, Visualization, Investigation, Formal analysis, Data curation. CD: Writing – review & editing, Visualization, Investigation, Formal analysis, Data curation. MW: Writing – review & editing, Investigation. ZL: Writing – review & editing, Investigation. EB: Writing – review & editing, Investigation, Formal analysis. CC: Writing – review & editing, Supervision, Project administration, Funding acquisition, Conceptualization.
