# Supplementary material for: CD8+ T cells are necessary for improved sepsis survival induced by CD28 agonism in immunologically experienced mice
Source: Front Immunol. 2024 Apr 3;15:1346097. doi: 10.3389/fimmu.2024.1346097 (PMC11021695; doi:10.3389/fimmu.2024.1346097)
Supplement: Supplementary file 1 [file DataSheet_1.pdf]

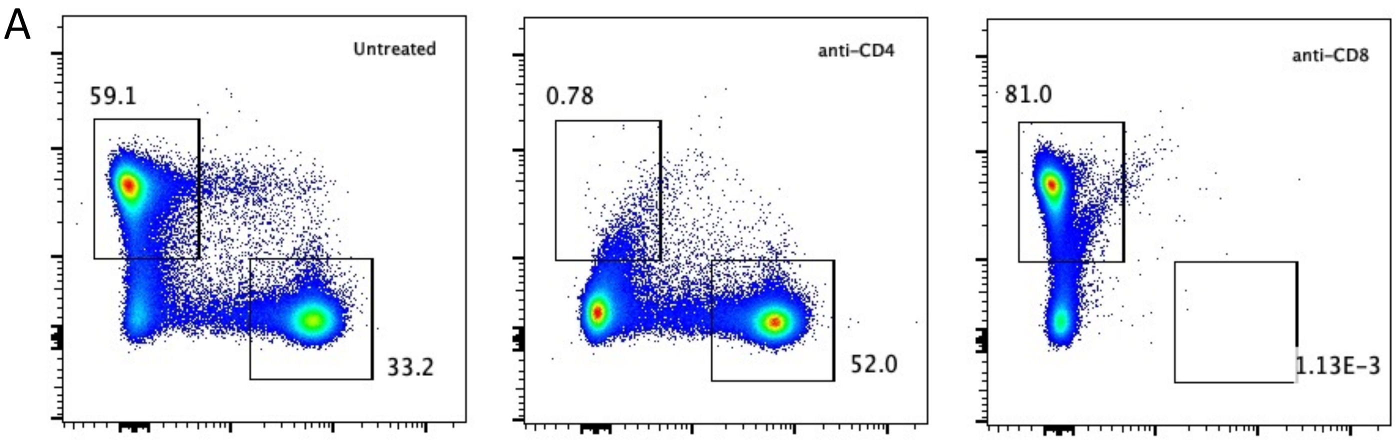

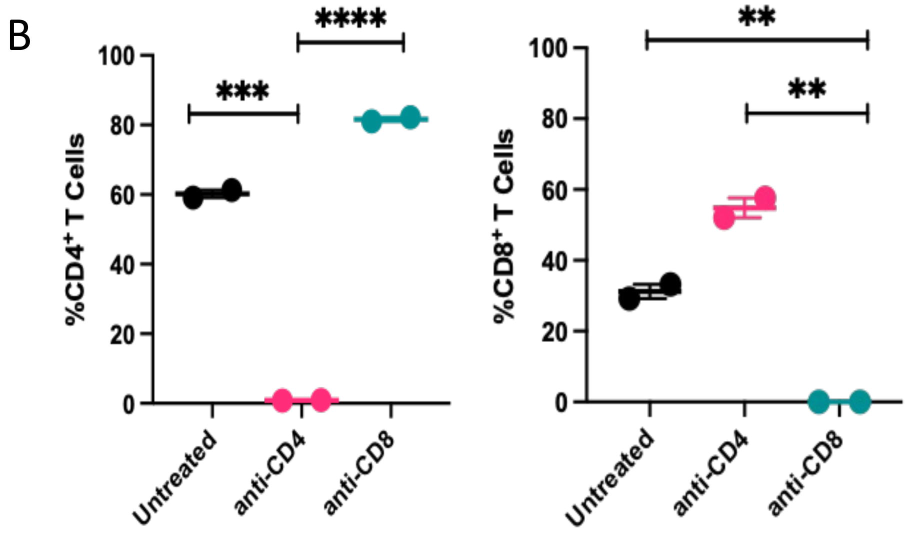

Supplemental Figure 1. Confirmation of depletion of CD4$^+$ and CD8$^+$ T cells in GK1.5 and YTS 196.4- treated animals, respectively. αCD4 antibody (GK1.5) or αCD8 antibody (clone YTS 196.4) were administered 24 hours prior to CLP and again 24 hours after CLP. Depletion was verified in the spleen at 24h.

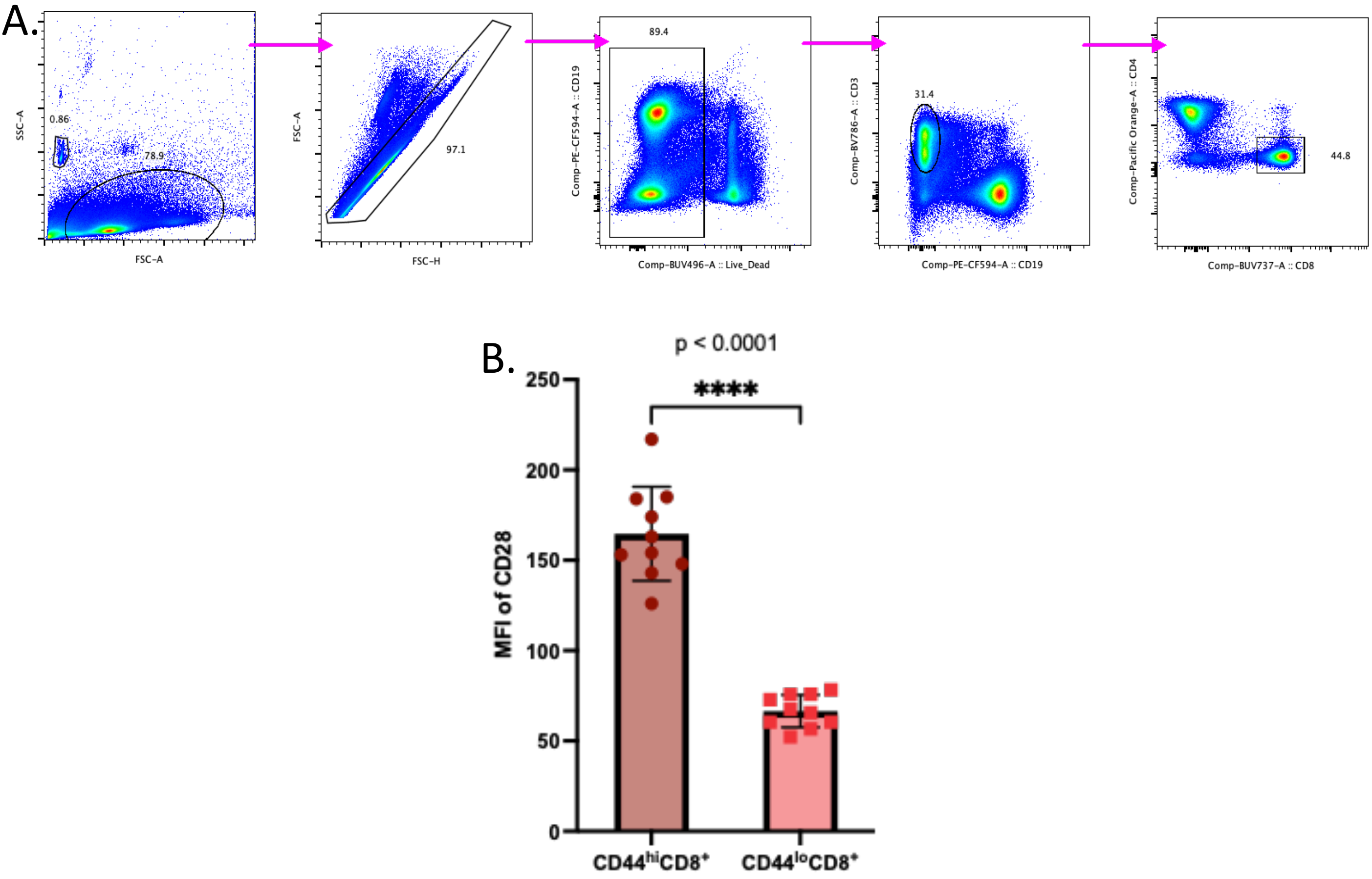

Supplemental Figure 2. **CD8$^+$ T cell gating strategy and expression of CD28.** Memory mice were subjected to CLP and the agonistic αCD28 antibody was given immediately following CLP and again on post-operative days 2, 4, and 6. Animals were sacrificed 24h later to assess CD8$^+$ T cells by flow cytometry. The gating strategy for these analyses in shown in A. B, Comparison of CD28 MFI between CD44^{hi} vs CD44^{lo} CD8$^+$ T cells isolated from vehicle control-treated memory septic mice at 24h post-CLP.

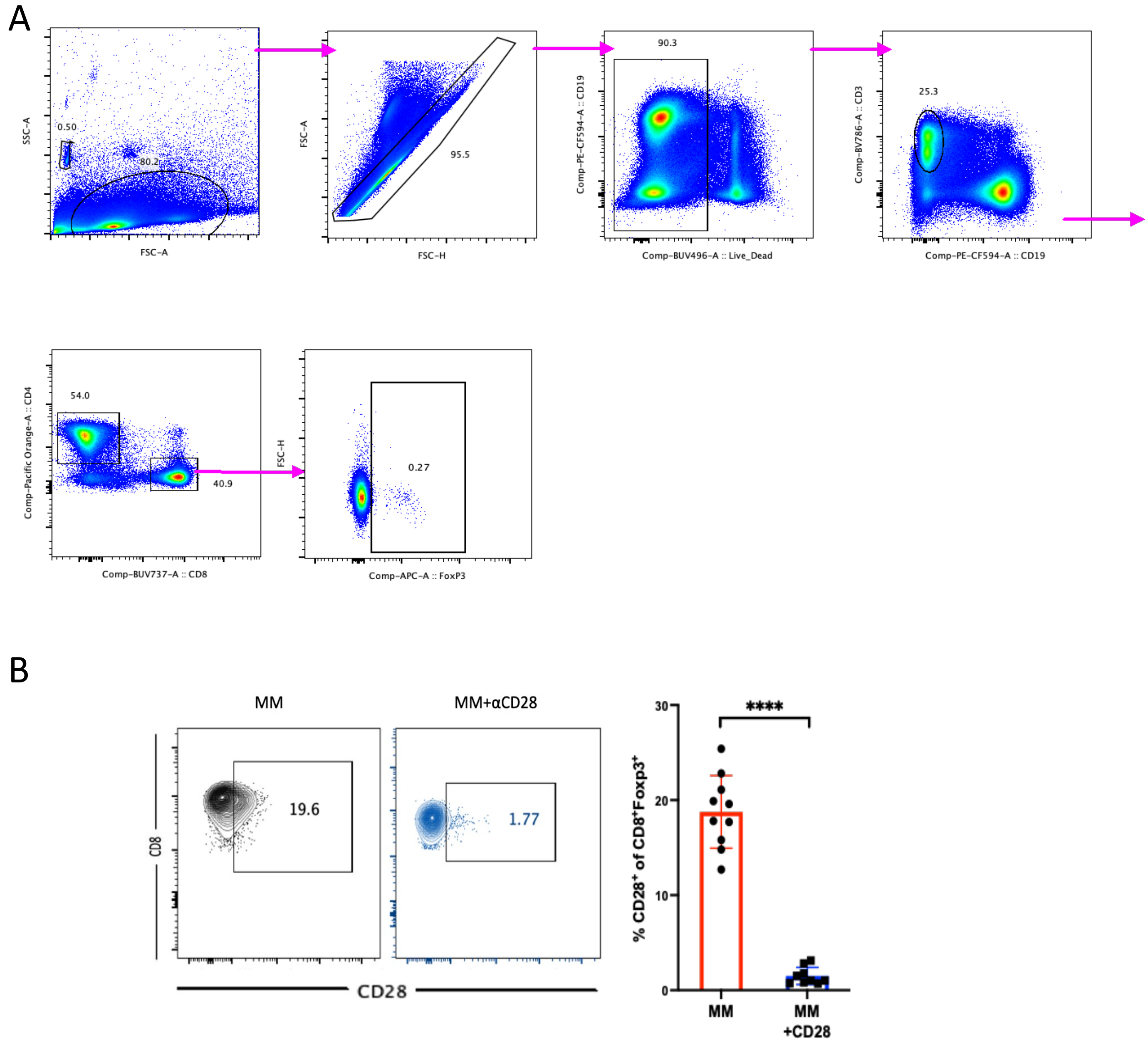

Supplemental Figure 3. Gating strategy and CD28 expression on Foxp3$^+$ CD8$^+$ T cells. Memory mice were subjected to CLP and the agonistic αCD28 antibody was given immediately following CLP and again on post-operative days 2, 4, and 6. Animals were sacrificed 24h later to assess Foxp3$^+$ CD8$^+$ T cells by flow cytometry. The gating strategy for these analyses in shown in A. B, Comparison of frequency of CD28+ cells among Foxp3$^+$ CD8$^+$ T cells isolated from vehicle control-treated vs. anti-CD28-treated memory septic mice at 24h post-CLP.
